# New secondary metabolites with cytotoxicity from fungus *Penicillium roqueforti*

**DOI:** 10.1007/s13659-023-00381-4

**Published:** 2023-06-01

**Authors:** Shuyuan Mo, Ziming Zhao, Zi Ye, Zhihong Huang, Yaxin Zhang, Wanqi Yang, Jianping Wang, Zhengxi Hu, Yonghui Zhang

**Affiliations:** Hubei Key Laboratory of Natural Medicinal Chemistry and Resource Evaluation, School of Pharmacy, Tongji Medical College, Huazhong University of Science and Technology, Wuhan, 430030 China

**Keywords:** *Hypericum beanii* N. Robson, Root soil-derived fungus, *Penicillium roqueforti*, Structural elucidation, Cytotoxicity

## Abstract

Two novel compounds including a cyclohelminthol type polyketide (namely oxaleimide K, **1**) and a maleimide derivative (namely peniroquefortine A, **2**), and a new natural product (namely 2-(acetylamino)-*N*-[(1*E*)-2-phenylethenyl]-acetamide, **3**), together with four known compounds (**4**–**7**), were isolated and identified from fungus *Penicillium roqueforti*, which was separated from the root soil of *Hypericum beanii* N. Robson collected from the Shennongjia Forestry District, Hubei Province. Their structures including absolute configurations were mainly established by the NMR spectroscopy analyses and single-crystal X-ray diffraction experiment. Compound **1** represents the second example of a cyclohelminthol type polyketide, which features a rare 6/6/5/5 tetracyclic system and a branched aliphatic chain containing a terminal olefin (oct-1-en-3-yl) moiety, and compound **2** possesses an unprecedented carbon skeleton that is uniquely defined by a maleimide moiety linked to the respective 4-methylene-2-(3-methylbut-2-en-1-yl)-phenol and *para*-substituted aromatic moieties via the carbon-carbon bonds. Remarkably, the absolute configuration of a cyclohelminthol type polyketide as exemplified by compound **1** is determined by the single-crystal diffraction analysis for the first time, highlighting an *E*-configuration for the linkage of a succinimide moiety and a tetrahydrofuran moiety for **1** rather than a *Z*-configuration as previously reported in the biosynthesis study, which gives a new insight into the structural elucidation of this category of polyketides. Additionally, compound **1** exhibited significant cytotoxic activity against multiple tumor cells, especially against the Farage and SU-DHL-2 cells (IC_50_ < 20 µM, 48 h). Further mechanism study revealed that compound **1** significantly induced cell cycle arrest in Farage and SU-DHL-2 cells by causing abnormal ROS level and triggering oxidative stress.

**Graphical Abstract:**

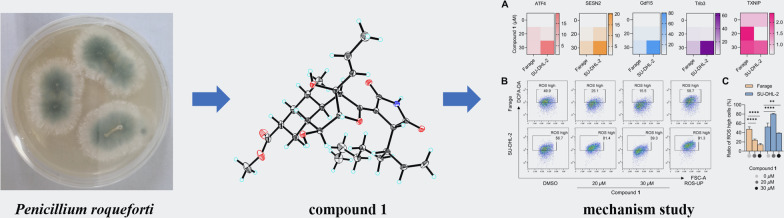

**Supplementary Information:**

The online version contains supplementary material available at 10.1007/s13659-023-00381-4.

## Introduction

Cancer is a life-threatening disease which has highly invasive and heterogeneous with different histological subtypes and mutation characteristics [1–3]. In 2020, research institutions revealed that there were 19.3 million cancer cases and nearly 10 million deaths worldwide, with time going by, the number will rapidly increase influenced by some factors such as the fast pace of life, high-fat diet, and so on [4]. As is known to all, microorganisms have played an indispensable role in drug development, and more than two-thirds of clinically used antitumor drugs are derived from natural products, such as taxol, romidepsin, salinomycin, and rapamycin et al. [5–8]. In recent decades, the increase in drug resistance, which caused the use of some antitumors greatly limited [9, 10]. Therefore, it is an emergency challenge to develop new antitumor agents with high efficacy and minimal side effects from microorganisms.

**Fig. 1:**
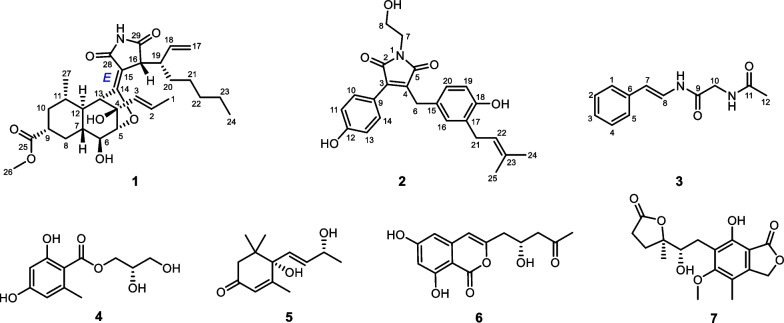
Chemical structures of compounds **1–7**

According to the extensive literature investigations, fungus *Penicilliu roqueforti* has been considered to be an irreplaceable resource of structurally diverse and bioactive natural products, including meroterpenoids, sesquiterpenoids, sesterterpenoids, alkaloids, etc., with attracting biological activities, such as antimicrobial, neurotoxic, and cytotoxic activities [11–13]. Therefore, strain *P. roqueforti* isolated from the root soil of *Hypericum beanii* N. Robson collected from the Shennongjia Forestry District of Hubei Province caught our attention and was systematically investigated, which led to the discovery of two novel secondary metabolites including a cyclohelminthol type polyketide (**1**) and a maleimide derivative (**2**), and a new natural product (**3**), as well as four known compounds (**4**–**7**). Compounds **1**–**7** were evaluated for the cytotoxic activities against six human tumor cell lines, including SU-DHL-2, RKO, A549, Jurkat, Farage, and HEPG2 cells, and the results revealed that compound **1** exhibited significant cytotoxic activity, especially against the Farage and SU-DHL-2 cells (IC_50_ < 20 *µ*M, 48 h). Herein, the isolation, structural elucidation, and bioactivities of these compounds are disclosed (Fig. 1).

## Results and discussion

Compound **1** was purified as a colorless block crystal. It was established by the HRESIMS ion peak at *m/z* 538.2791 ([M + Na]^+^, calcd for 538.2775), which suggested a molecular formula of C_29_H_41_NO_7_ with 10 degrees of unsaturation. Conventional ^13^C NMR spectroscopy analyses (Table 1) of **1** revealed the presence of twenty-nine carbon resonances including three methyls (*δ*_C_ 21.1, 18.1, and 14.4), one methoxy (*δ*_C_ 52.1), seven methylenes (*δ*_C_ 116.7, 40.9, 35.3, 33.0, 31.5, 28.4, and 23.7), twelve methines (*δ*_C_ 140.9, 135.0, 127.3, 87.1, 75.8, 53.3, 49.8, 46.4, 45.6, 44.6, 43.3, and 38.5), and six non-protonated carbons (*δ*_C_ 179.7, 177.4, 173.8, 172.4, 99.7, and 79.8). Through extensive literature research and analysis of aforementioned data, compound **1** was deduced to be a structural analogue of cyclohelminthol CP-1 [14]. The three double bonds, one ester carbonyl, and two amide carbonyls accounted for six out of 10 degrees of unsaturation, implying that **1** possessed a tetracyclic system (Additional files 1, 2).

**Table 1:** ^1^H NMR and ^13^C NMR spectroscopic data (*δ* in ppm, *J* in Hz) for **1**–**3**

No.	**1**		**2**		**3**	
	*δ*_C_^a,c^	*δ*_H_^a,b^	*δ*_C_^a,e^	*δ*_H_^a,d^	*δ*^a,c^	*δ*_H_^a,b^
1	18.1 CH_3_	1.70 dd (6.5, 1.7)			126.5 CH	7.32 m
2	127.3 CH	5.81 dq (15.6, 6.5)	173.1 C		129.7 CH	7.27 dd (8.5, 7.0)
3	135.0 CH	5.59 dq (15.6, 1.7)	138.9 C		127.7 CH	7.16 m
4	79.8 C		137.3 C		129.7 CH	7.27 dd (8.5, 7.0)
5	87.1 CH	4.10 s	173.8 C		126.5 CH	7.32 m
6	75.8 CH	3.50 d (9.7)	29.7 CH_2_	3.77 s	137.8 C	
7	43.3 CH	2.62 m	41.4 CH_2_	3.65 m	115.0 CH	6.26 d (14.7)
8	35.3 CH_2_	2.35 m; 1.10 m	60.3 CH_2_	3.67 m	123.5 CH	7.45 d (14.7)
9	44.6 CH	2.55 m	121.2 C		169.3 C	
10	40.9 CH_2_	2.06 m; 1.15 m	132.4 CH	7.43 d (8.7)	43.5 CH_2_	3.93 s
11	38.5 CH	1.74 m	116.6 CH	6.79 d (8.7)	173.9 C	
12	53.3 CH	1.04 t (12.2)	161.0 C		22.4 CH_3_	2.03 s
13	45.6 CH	3.84 s	116.6 CH	6.79 d (8.7)		
14	173.8 C		132.4 CH	7.43 d (8.7)		
15	99.7 C		129.6 C			
16	49.8 CH	3.36 d (3.4)	130.5 CH	6.81 s		
17	116.7 CH_2_	5.07 dd (17.1, 1.9); 5.05 dd (10.1, 1.9)	129.4 C			
18	140.9 CH	5.90 ddd (17.1, 10.1, 9.3)	154.8 C			
19	46.4 CH	2.72 m	115.9 CH	6.61 d (8.2)		
20	31.5 CH_2_	1.40 m; 1.32 m	127.6 CH	6.79 d (8.2)		
21	28.4 CH_2_	1.40 m; 1.24 m	29.0 CH_2_	3.17 d (7.5)		
22	33.0 CH_2_	1.32 m; 1.24 m	123.7 CH	5.21 m		
23	23.7 CH_2_	1.32 m	133.2 C			
24	14.4 CH_3_	0.91 t (7.1)	17.8 CH_3_	1.62 s		
25	177.4 C		26.0 CH_3_	1.68 s		
26	52.1 OCH_3_	3.67 s				
27	21.1 CH_3_	1.20 d (6.5)				
28	172.4 C					
29	179.7 C					

^a^In methanol-*d*_4_

^b^Recorded at 600 MHz

^c^Recorded at 150 MHz

^d^Recorded at 400 MHz

^e^Recorded at 100 MHz

“m” means overlapped or multiplet with other signals

**Fig. 2:**
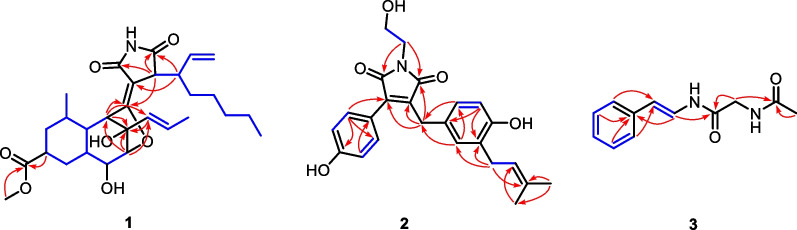
Key ^1^H–^1^H COSY (blue lines) and HMBC (red arrows) correlations of compounds **1**–**3**

**Fig. 3:**
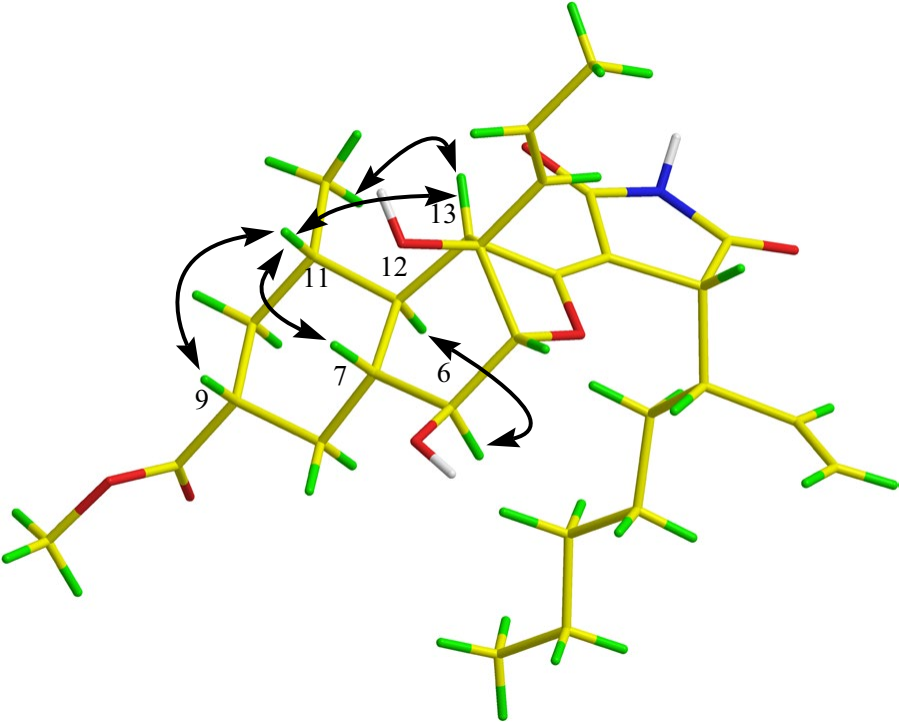
Key ROESY correlations (black arrows) of compound **1**

**Fig. 4:**
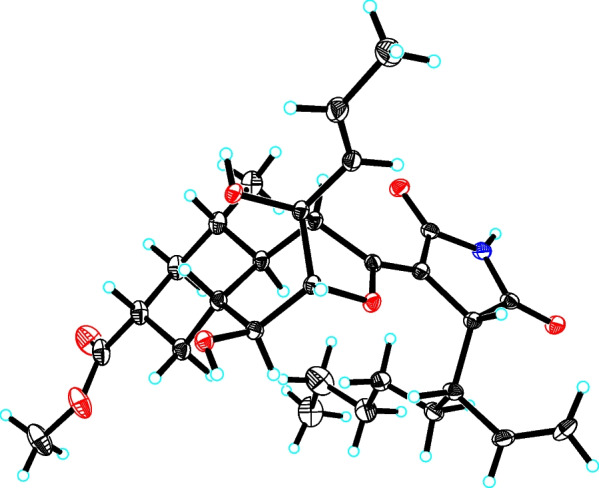
X-ray crystallographic structure of **1**

The 1D NMR data (Table 1) of **1** were closely similar to those of the known compound cyclohelminthol CP-1 [14], suggesting that both compounds shared the same core skeleton, and the differences were shown as follows: (1) a –COOH group at C-9 in cyclohelminthol CP-1 was replaced by a –COOCH_3_ group at the same position in **1**; (2) a C-17/C-22 hexyl side chain linked at C-16 in cyclohelminthol CP-1 was replaced by a branched aliphatic chain containing a terminal olefin (oct-1-en-3-yl) moiety linked at the same position in **1**, which were fully supported by the key ^1^H–^1^H COSY correlations of H_3_-24/H_2_-23/H_2_-22/H_2_-21/H_2_-20/H-19 (H-16)/H-18/H_2_-17 and HMBC correlations from H-19 to C-15/C-29 and from H-9/H_3_-26 to C-25 (Fig. 2). The relative configuration of **1** was partially verified by analyzing the ROESY data. The ROESY correlations (Fig. 3) from H-11 to H-7/H-9 determined that these protons were on the same side with *β*-orientations, while the ROESY cross-peak (Fig. 3) of H-12 to H-6 suggested that H-12 and H-6 were on the other side. However, the configurations of C-4, C-5, C-6, C-13, and C-19 could not be determined by analyzing the NMR data alone. Luckily, a crystal of **1** suitable for X-ray diffraction crystallographic analysis was furnished, which unequivocally confirmed the absolute configuration of **1** as 4*R*,5*R*,6*S*,7*R*,9*R*,11*S*,12*S*,13*R*,16*R*,19*S* with Cu K*α* (Fig. 4) [Flack parameter = 0.02(5), CCDC 2,238,093]. Accordingly, the absolute structure of **1** was defined.

Compound **2**, obtained as a yellow oil, was established a molecular formula of C_24_H_25_NO_5_ on the basis of the positive HRESIMS ion peak at *m/z* 430.1631 [M + Na]^+^ (calcd for C_24_H_25_NO_5_Na^+^, 430.1625) in the HRESIMS spectrum, suggesting 13 degrees of unsaturation. The IR spectrum of **1** showed obvious absorptions for a hydroxy group (3442 cm^−1^) and an amide carbonyl group (1698 cm^−1^). The ^1^H NMR data (Table 1) indicated characteristic signals for an alkenyl proton at *δ*_H_ 5.21 (H-22), two methyl groups at *δ*_H_ 1.62 (H-24) and 1.68 (H-25), two sets of *para*-substituted aromatic protons at *δ*_H_ 7.43 (H-10/H-14) and 6.79 (H-11/H-13), and 1,2,4-trisubstituted aromatic protons at *δ*_H_ 6.81 (H-16), 6.61 (H-19), and 6.79 (H-20). The ^13^ C NMR data (Table 1) displayed the presence of 24 carbon resonances including two methyls (*δ*_C_ 26.0 and 17.8), four methylenes (*δ*_C_ 60.3, 41.4, 29.7, and 29.0), eight methines (*δ*_C_ 132.4, 132.4, 130.5, 127.6, 123.7, 116.6, 116.6, and 115.9), and ten non-protonated carbons (*δ*_C_ 173.8, 173.1, 161.0, 154.8, 138.9, 137.3, 133.2, 129.6, 129.4, and 121.2). Considering the degree of unsaturation, we could draw a conclusion that compound **2** was a maleimide derivative with a tricyclic system. By comparing the above spectral data and extensive literature research, the structure of **2** was somewhat similar to the reported compound 1-hydroxy-3-(4-hydroxyphenyl)-4-isobutyl-1* H*-pyrrole-2,5-dione [15]. The differences were shown as follows: (1) the appearance of a 4-methylene-2-(3-methylbut-2-en-1-yl)-phenol moiety connected at C-4 in compound **2**, which could be proved by the HMBC correlations (Fig. 2) from H_2_-6 to C-3/C-4/C-5 and from H-16/H-20 to C-6; (2) the existence of a -CH_2_CH_2_OH moiety linked at *N*-1 in compound **2**, which was supported by the HMBC correlations (Fig. 2) from H_2_-7 to C-2/C-5. Accordingly, the structure of **2** was defined.

Compound **3** possessed a molecular formula of C_12_H_14_N_2_O_2_ as determined by the HRESIMS and NMR data, indicating seven degrees of unsaturation. The ^1^H NMR data (Table 1) exhibited signals for a mono-substituted phenyl ring at *δ*_H_ 7.32 (H-1/H-5), 7.27 (H-2/H-4), and 7.16 (H-3), one methyl at *δ*_H_ 2.03 (H-12), one methylene at *δ*_H_ 3.93 (H-10), and two alkenyl protons at *δ*_H_ 7.45 (H-8) and *δ*_H_ 6.26 (H-7). The ^13^C NMR data (Table 1) displayed twelve carbon resonances that were assigned to six phenyl C-atoms (*δ*_C_ 137.8, 129.7, 129.7, 127.7, 126.5, and 126.5), two olefinic C-atoms (*δ*_C_ 129.7 and 127.7), two amide C-atoms (*δ*_C_ 173.9 and 169.3), one methylene C-atom (*δ*_C_ 43.5), and one methyl C-atom (*δ*_C_ 22.4). Detailed 2D NMR data analyses (Fig. 2) designated the planar structure of **3**, which was the same as a known synthetic product 2-(acetylamino)-*N*-[(1*E*)-2-phenylethenyl]-acetamide [16]. Accordingly, the structure of **3** was assigned to be a new natural product.

The four known compounds, compared with the literature, were identified as hydroxypropan-2',3'-diol orsellinate (**4**) [17], vomifoliol (**5**) [18], citreoisocoumarin (**6**) [19], and (–)-brevicolide A (**7**) [20].

**Fig. 5:**
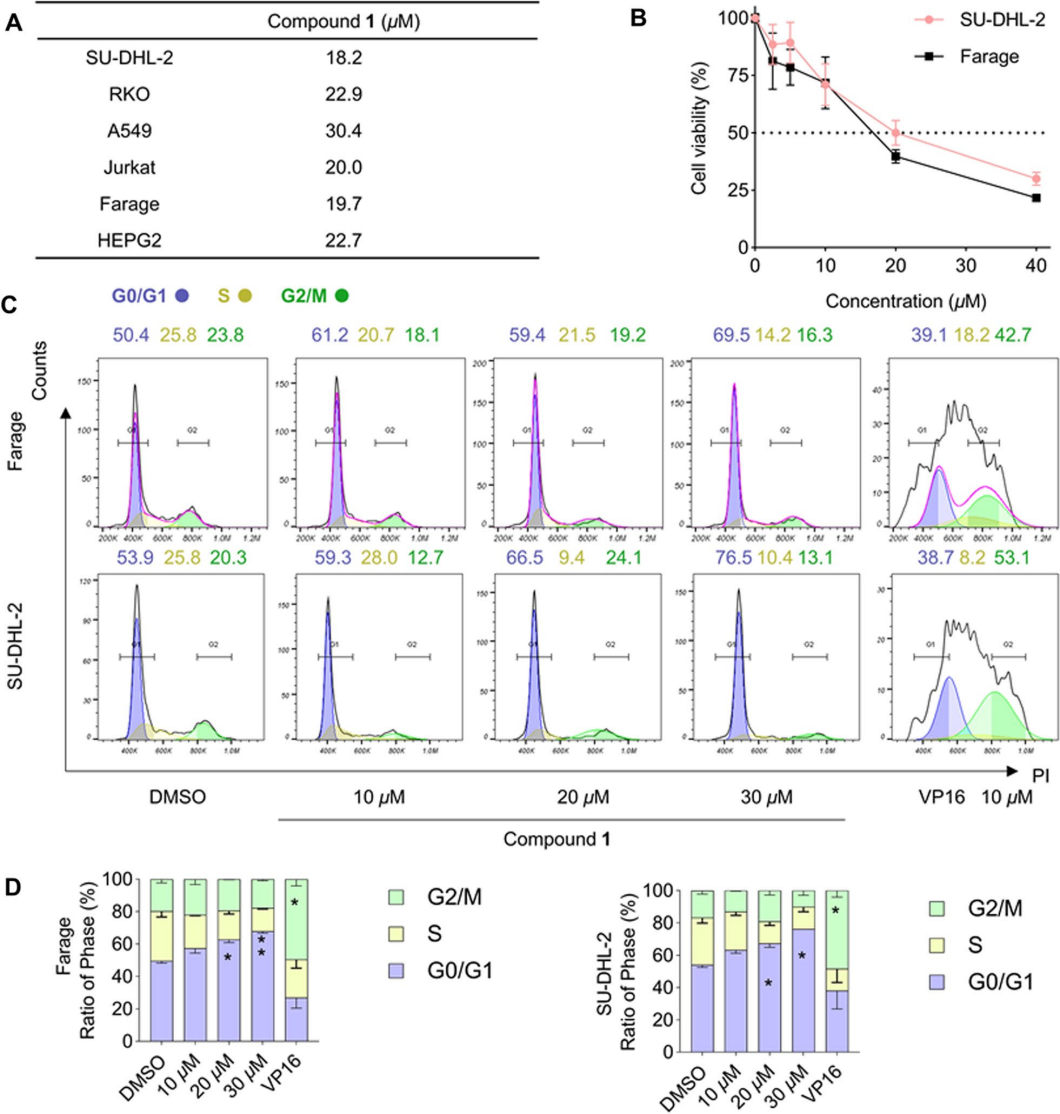
Compound **1** blocked the cell cycle to exert antitumor effects. **A** IC_50_ calculated by cell viability assays against six human tumor cells exposed to the increasing doses of compound **1** over the course of 48 h. Mean ± s.d., n = 3. **B** Cell viability assays against the Farage and SU-DHL-2 cells exposed to the increasing doses of compound **1** over the course of 48 h. Mean ± s.d., n = 3. **C**–**D** Cell cycle assays against the Farage and SU-DHL-2 cell lines exposed to the increasing doses of compound **1** (10 *~* 30 µM) or VP16 over the course of 24 h. Mean ± s.d., n = 3 (*p < 0.05, **p < 0.01, ***p < 0.001, ****p < 0.0001, compared to the control group DMSO)

**Fig. 6:**
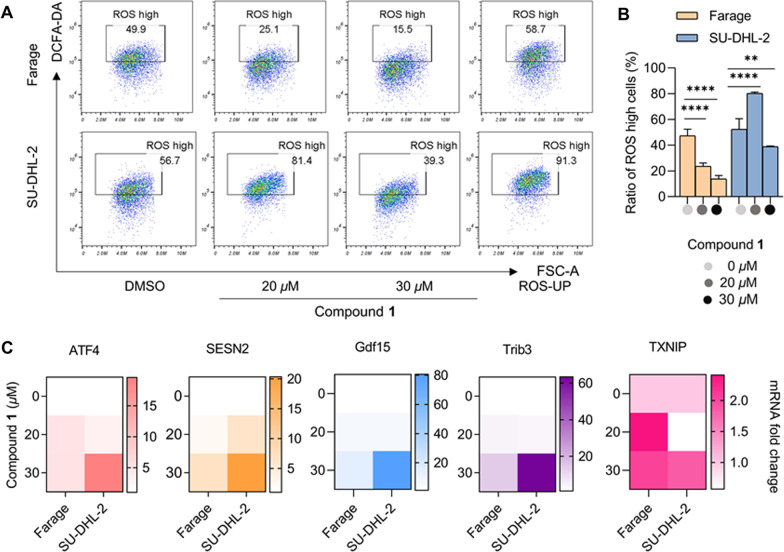
Compound **1** decreased the intracellular ROS level and triggered oxidative stress in tumor cells. **A**–**B** Intracellular ROS assays in Farage and SU-DHL-2 cells exposed to the increasing doses of compound **1** over the course of 24 h. Mean ± s.d., n = 3. **C** Oxidative stress-related gene mRNA analysis in Farage and SU-DHL-2 cells exposed to compound **1** (20 or 30 µM, 24 h, n = 3)

We evaluated the cytotoxic activities of compounds **1**–**7** against six human tumor cell lines, including SU-DHL-2 (Diffuse large B-cell lymphoma), RKO (Colonic adenocareinoma), A549 (Lung cancer), Jurkat (T cell acute leukemia), Farage (Diffuse large B-cell lymphoma), and HEPG2 (Hepatocellular carcinoma). As a result, only compound **1** exhibited significant cytotoxic activity against multiple tumor cells, especially against the Farage and SU-DHL-2 (IC_50_ < 20 *µ*M, 48 h) cells (Fig. 5A–B). Subsequently, we examined whether compound **1** could cause cell cycle arrest in Farage and SU-DHL-2 cells at a series of concentrations of 10, 20, and 30 *µ*M, and the positive drug VP16 (Etoposide, 10 *µ*M) concentration gradient for 24 h. The above results showed that compound **1** could significantly block G0/G1 phase (the stationary and pre-synthetic phase of the cell cycle) in both Farage and SU-DHL-2 cells, and the cell cycle arrest was dose-dependent (Fig. 5C–D). It is well known that tumor cells have a unique metabolic pattern and the highly activated oxidative metabolism in tumor cells makes them sensitive to ROS-induced oxidative stress, and abnormal ROS levels can lead to tumor cell cycle arrest or apoptosis [21, 22]. Therefore, we suspected that compound **1** exerted its antitumor activity by regulating ROS levels in Farage and SU-DHL-2 cells. Meanwhile, for the sake of discovering whether compound **1** had the ability to regulate ROS levels, we detected the level of oxidative stress in compound **1**-treated tumor cells. As shown in Fig. 6A–B, in the more sensitive Farage cells, compound **1** significantly attenuated cellular ROS levels, which might represent an inhibition of cellular respiration or a state of frequent cell death. In contrast, in the SU-DHL-2 cells, low-dose treatment of compound **1** induced a significant increase of ROS levels, while high-dose treatment of compound **1** significantly attenuated cellular ROS levels. Consequently, we speculated that the disruption of redox homeostasis in SU-DHL-2 cells by compound **1** involved a dynamic process distinct from that in Farage cells, which ROS first rose and then fell, with elevated ROS inducing cellular oxidative stress and cell damage, followed by a decrease in ROS levels below normal levels and impaired proliferation. Finally, we examined the expression levels of some genes mRNA related to oxidative stress. The heat map showed that the expression levels of ATF4, SESN2, Gdf15, Trib3, and TXNIP were highly up-regulated after compound **1**-treatment, and the cells were in a significant oxidative stress state after compound **1**-treatment (Fig. 6C). Overall, we found that compound **1** significantly induced cell cycle arrest in Farage and SU-DHL-2 cells by causing abnormal ROS levels and triggering oxidative stress, which can be further investigated as a clinical candidate lead compound for treating tumor-related diseases.

The chemical investigation on fungus *P. roqueforti* afforded two novel compounds including a cyclohelminthol type polyketide (**1**) and a maleimide derivative (**2**), and a new natural product (**3**), as well as four known compounds (**4**–**7**). Compound **1** represents the second example of a cyclohelminthol type polyketide, which features a rare 6/6/5/5 tetracyclic system and a branched aliphatic chain containing a terminal olefin (oct-1-en-3-yl) moiety, and compound **2** possesses an unprecedented carbon skeleton that is uniquely defined by a maleimide moiety linked with the respective 4-methylene-2-(3-methylbut-2-en-1-yl)-phenol and *para*-substituted aromatic moieties via the carbon-carbon bonds. Compound **1** showed significant cytotoxic activity against the Farage and SU-DHL-2 cells, which induced apoptosis in a dose-dependent manner and cell growth arrest at the G0/G1 phase. The mechanism study revealed that compound **1** could regulate the ROS level in tumor cells, thereby inducing oxidative stress and cell damage and destroying the redox homeostasis. This work provides new insights into the chemical diversity of fungus *P. roqueforti*, and highlights compound **1** as a potential clinical antitumor candidate.

## Supplementary Information

**Additional file 1:  Figure S1. **^1^H NMR spectrum of compound **1** (Recorded in methanol-*d*_4_). **Figure S2**. ^13^C NMR and DEPT spectra of compound **1** (Recorded in methanol-*d*_4_). **Figure S3**. HSQC spectrum of compound **1** (Recorded in methanol-*d*_4_). **Figure S4**. HMBC spectrum of compound **1** (Recorded in methanol-*d*_4_). **Figure S5**. ^1^H–^1^H COSY spectrum of compound **1** (Recorded in methanol-*d*_4_). **Figure S6**. ROESY spectrum of compound **1** (Recorded in methanol-*d*_4_). **Figure S7**. HRESIMS spectrum of compound **1**. **Figure S8**. UV spectrum of compound **1**. **Figure S9**. IR spectrum of compound **1**. **Figure S10**. ECD spectrum of compound **1**. **Figure S11**. ^1^H NMR spectrum of compound **2** (Recorded in methanol-*d*_4_). **Figure S12**. ^13^C NMR and DEPT spectra of compound **2** (Recorded in methanol-*d*_4_). **Figure S13**. HSQC spectrum of compound **2** (Recorded in methanol-*d*_4_). **Figure S14**. HMBC spectrum of compound **2** (Recorded in methanol-*d*_4_). **Figure S15**. ^1^H–^1^HCOSY spectrum of compound **2** (Recorded in methanol-*d*_4_). **Figure S16**. NOESY spectrum of compound **2** (Recorded in methanol-*d*_4_). **Figure S17**. HRESIMS spectrum of compound **2**. **Figure S18**. UV spectrum of compound **2**. **Figure S19**. IR spectrum of compound **2**. **Figure S20**. ECD spectrum of compound **2**. **Figure S21**. ^1^H NMR spectrum of compound **3** (Recorded in methanol-*d*_4_). **Figure S22**. ^13^C NMR and DEPT spectra of compound **3** (Recorded in methanol-*d*_4_). **Figure S23**. HSQC spectrum of compound **3** (Recorded in methanol-*d*_4_). **Figure S24**. HMBC spectrum of compound **3** (Recorded in methanol-*d*_4_). **Figure S25**. ^1^H–^1^H COSY spectrum of compound **3** (Recorded in methanol-*d*_4_). **Figure S26**. ROESY spectrum of compound **3** (Recorded in methanol-*d*_4_). **Figure S27**. HRESIMS spectrum of compound **3**. **Figure S28**. UV spectrum of compound **3**. **Figure S29**. IR spectrum of compound **3**. **Figure S30**. ECD spectrum of compound **3**.

**Additional file 2**: Additional data associated with this article including 1D and 2D NMR, HRESIMS, UV, IR of **1**–**3** and X-ray data of compound **1**.
