# Supplementary material for: New secondary metabolites with cytotoxicity from fungus Penicillium roqueforti
Source: Nat Prod Bioprospect. 2023 Jun 1;13(1):17. doi: 10.1007/s13659-023-00381-4 (PMC10235286; doi:10.1007/s13659-023-00381-4)
Supplement: Supplementary file 1 — Additional file 1: Figure S1. 1H NMR spectrum of compound 1 (Recorded in methanol-d4). Figure S2. 13C NMR and DEPT spectra of compound 1 (Recorded in methanol-d4). Figure S3. HSQC spectrum of compound 1 (Recorded in methanol-d4). Figure S4. HMBC spectrum of compound 1 (Recorded in methanol-d4). Figure S5. 1H–1H COSY spectrum of compound 1 (Recorded in methanol-d4). Figure S6. ROESY spectrum of compound 1 (Recorded in methanol-d4). Figure S7. HRESIMS spectrum of compound 1. Figure S8. UV spectrum of compound 1. Figure S9. IR spectrum of compound 1. Figure S10. ECD spectrum of compound 1. Figure S11. 1H NMR spectrum of compound 2 (Recorded in methanol-d4). Figure S12. 13C NMR and DEPT spectra of compound 2 (Recorded in methanol-d4). Figure S13. HSQC spectrum of compound 2 (Recorded in methanol-d4). Figure S14. HMBC spectrum of compound 2 (Recorded in methanol-d4). Figure S15. 1H–1HCOSY spectrum of compound 2 (Recorded in methanol-d4). Figure S16. NOESY spectrum of compound 2 (Recorded in methanol-d4). Figure S17. HRESIMS spectrum of compound 2. Figure S18. UV spectrum of compound 2. Figure S19. IR spectrum of compound 2. Figure S20. ECD spectrum of compound 2. Figure S21. 1H NMR spectrum of compound 3 (Recorded in methanol-d4). Figure S22. 13C NMR and DEPT spectra of compound 3 (Recorded in methanol-d4). Figure S23. HSQC spectrum of compound 3 (Recorded in methanol-d4). Figure S24. HMBC spectrum of compound 3 (Recorded in methanol-d4). Figure S25. 1H–1H COSY spectrum of compound 3 (Recorded in methanol-d4). Figure S26. ROESY spectrum of compound 3 (Recorded in methanol-d4). Figure S27. HRESIMS spectrum of compound 3. Figure S28. UV spectrum of compound 3. Figure S29. IR spectrum of compound 3. Figure S30. ECD spectrum of compound 3. [file 13659_2023_381_MOESM1_ESM.doc]

Additional file 1

**New secondary metabolites with cytotoxicity from** **fungus** ***Penicillium roqueforti***

Shuyuan Mo^1^, Ziming Zhao^1^, Zi Ye, Zhihong Huang, Yaxin Zhang, Wanqi Yang, Jianping Wang*, Zhengxi Hu* and Yonghui Zhang*

*Hubei Key Laboratory of Natural Medicinal Chemistry and Resource Evaluation,* *School of Pharmacy, Tongji Medical College, Huazhong University of Science and Technology, Wuhan 430030, China*

* Corresponding author Tel.: 86-27-83692892

E-mail: zhangyh@mails.tjmu.edu.cn (Y. Zhang); hzx616@126.com (Z. Hu); jpwang1001@163.com

(J. Wang)

**Contents of Additional file 1**

[**Figure S1.** ^1^H NMR spectrum of compound **1** (Recorded in methanol-*d*_4_) 3](#__RefHeading___Toc126958970)

[**Figure S2.** ^13^C NMR and DEPT spectra of compound **1** (Recorded in methanol-*d*_4_) 4](#__RefHeading___Toc126958971)

[**Figure S3.** HSQC spectrum of compound **1** (Recorded in methanol-*d*_4_) 5](#__RefHeading___Toc126958972)

[**Figure S4.** HMBC spectrum of compound **1** (Recorded in methanol-*d*_4_) 6](#__RefHeading___Toc126958973)

[**Figure S5.** ^1^H–^1^H COSY spectrum of compound **1** (Recorded in methanol-*d*_4_) 7](#__RefHeading___Toc126958974)

[**Figure S6.** NOESY spectrum of compound **1** (Recorded in methanol-*d*_4_) 8](#__RefHeading___Toc126958975)

[**Figure S7.** HRESIMS spectrum of compound **1** 9](#__RefHeading___Toc126958976)

[**Figure S8.** UV spectrum of compound **1** 10](#__RefHeading___Toc126958977)

[**Figure S9.** IR spectrum of compound **1** 11](#__RefHeading___Toc126958978)

[**Figure S10.** ECD spectrum of compound **1** 12](#__RefHeading___Toc126958979)

[**Figure S11.** ^1^H NMR spectrum of compound **2** (Recorded in methanol-*d*_4_) 13](#__RefHeading___Toc126958981)

[**Figure S12.** ^13^C NMR and DEPT spectra of compound **2** (Recorded in methanol-*d*_4_) 14](#__RefHeading___Toc126958982)

[**Figure S13.** HSQC spectrum of compound **2** (Recorded in methanol-*d*_4_) 15](#__RefHeading___Toc126958983)

[**Figure S14.** HMBC spectrum of compound **2** (Recorded in methanol-*d*_4_) 16](#__RefHeading___Toc126958984)

[**Figure S15.** ^1^H–^1^H COSY spectrum of compound **2** (Recorded in methanol-*d*_4_) 17](#__RefHeading___Toc126958985)

[**Figure S16.** NOESY spectrum of compound **2** (Recorded in methanol-*d*_4_) 18](#__RefHeading___Toc126958986)

[**Figure S17.** HRESIMS spectrum of compound **2** 19](#__RefHeading___Toc126958987)

[**Figure S18.** UV spectrum of compound **2** 20](#__RefHeading___Toc126958988)

[**Figure S19.** IR spectrum of compound **2** 21](#__RefHeading___Toc126958989)

[**Figure S20.** ECD spectrum of compound **2** 22](#__RefHeading___Toc126958990)

[**Figure S21.** ^1^H NMR spectrum of compound **3** (Recorded in methanol-*d*_4_) 23](#__RefHeading___Toc126958991)

[**Figure S22.** ^13^C NMR and DEPT spectra of compound **3** (Recorded in methanol-*d*_4_) 24](#__RefHeading___Toc126958992)

[**Figure S23.** HSQC spectrum of compound **3** (Recorded in methanol-*d*_4_) 25](#__RefHeading___Toc126958993)

[**Figure S24.** HMBC spectrum of compound **3** (Recorded in methanol-*d*_4_) 26](#__RefHeading___Toc126958994)

[**Figure S25.** ^1^H–^1^H COSY spectrum of compound **3** (Recorded in methanol-*d*_4_) 27](#__RefHeading___Toc126958995)

[**Figure S26.** NOESY spectrum of compound **3** (Recorded in methanol-*d*_4_) 28](#__RefHeading___Toc126958996)

[**Figure S27.** HRESIMS spectrum of compound **3** 29](#__RefHeading___Toc126958997)

[**Figure S28.** UV spectrum of compound **3** 30](#__RefHeading___Toc126958998)

[**Figure S29.** IR spectrum of compound **3** 31](#__RefHeading___Toc126958999)

[**Figure S30.** ECD spectrum of compound **3** 32](#__RefHeading___Toc126959000)

[**Experimental** 33](#__RefHeading___Toc101278714)

[**Cytotoxicity and cell apoptosis assay** 36](#__RefHeading___Toc101278714)

**Figure S1.** ^1^H NMR spectrum of compound **1** (Recorded in methanol-*d*_4_)

**Figure S2.** ^13^C NMR and DEPT spectra of compound **1** (Recorded in methanol-*d*_4_)

**Figure S3.** HSQC spectrum of compound **1** (Recorded in methanol-*d*_4_)

**Figure S4.** HMBC spectrum of compound **1** (Recorded in methanol-*d*_4_)

**Figure S5.** ^1^H–^1^H COSY spectrum of compound **1** (Recorded in methanol-*d*_4_)

**Figure S6.** NOESY spectrum of compound **1** (Recorded in methanol-*d*_4_)

**Figure S7.** HRESIMS spectrum of compound **1**

**Figure S8.** UV spectrum of compound **1**


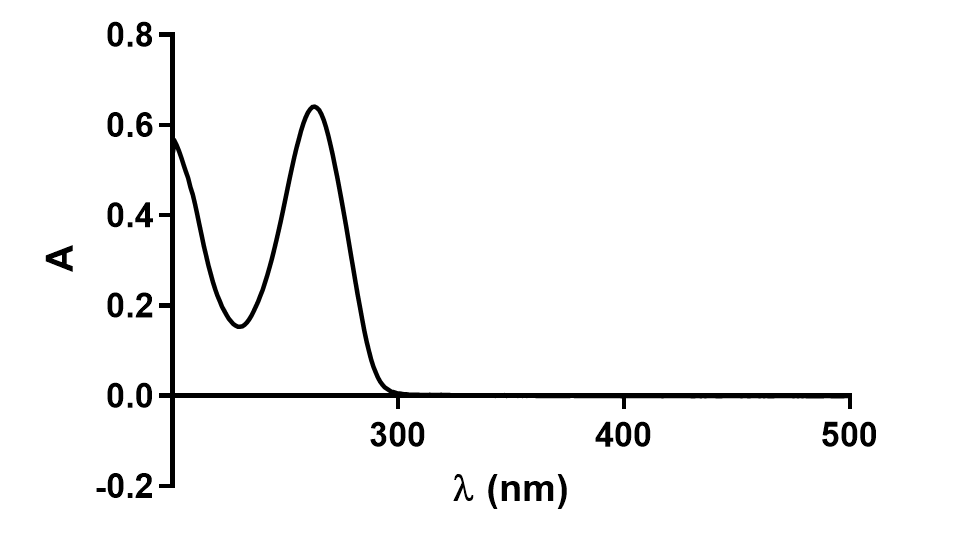


**Figure S9.** IR spectrum of compound **1**

**Figure S10.** ECD spectrum of compound **1**

**
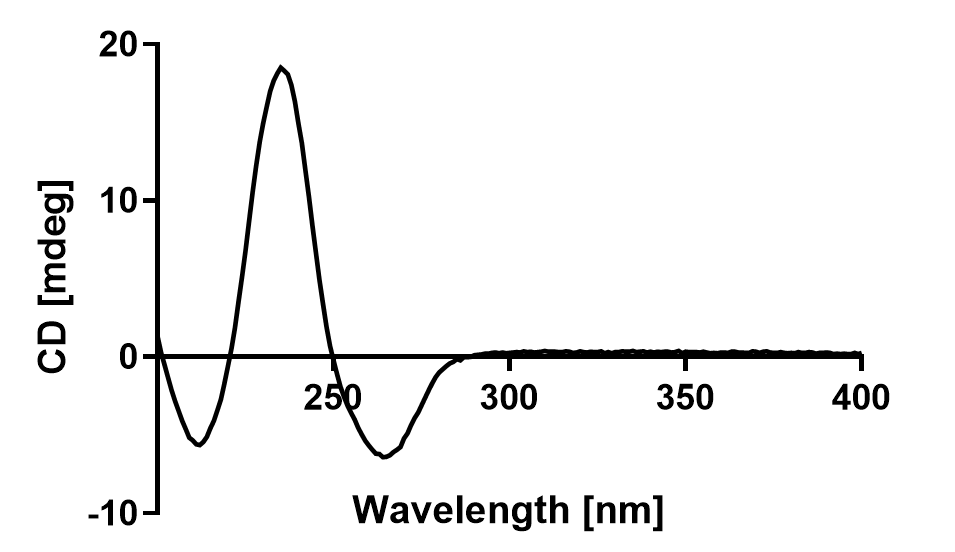
**

**Figure S11.** ^1^H NMR spectrum of compound **2** (Recorded in methanol-*d*_4_)

**Figure S12.** ^13^C NMR and DEPT spectra of compound **2** (Recorded in methanol-*d*_4_)

**Figure S13.** HSQC spectrum of compound **2** (Recorded in methanol-*d*_4_)

**Figure S14.** HMBC spectrum of compound **2** (Recorded in methanol-*d*_4_)

**Figure S15.** ^1^H–^1^H COSY spectrum of compound **2** (Recorded in methanol-*d*_4_)

**Figure S16.** NOESY spectrum of compound **2** (Recorded in methanol-*d*_4_)

**Figure S17.** HRESIMS spectrum of compound **2**

**Figure S18.** UV spectrum of compound **2**


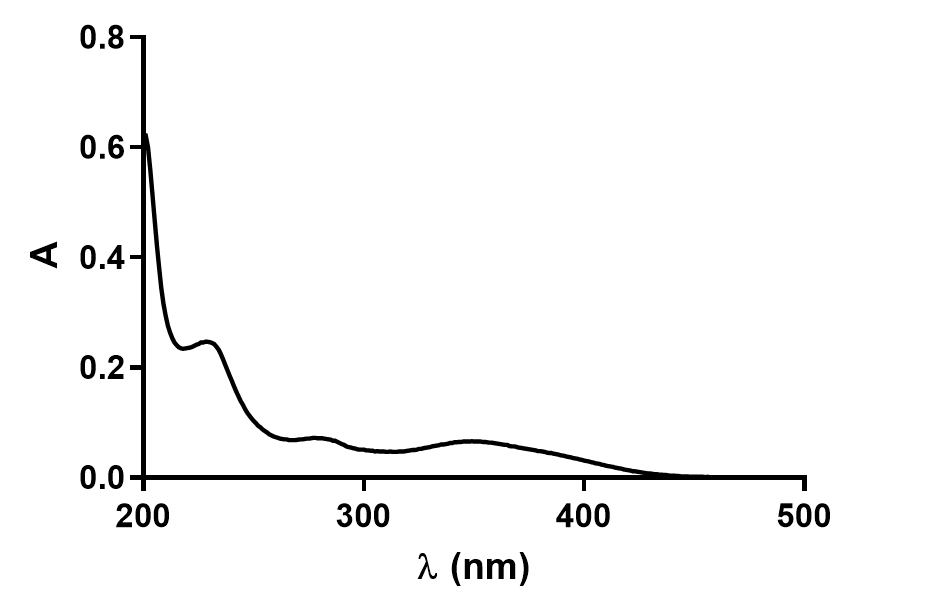


**Figure S19.** IR spectrum of compound **2**

**Figure S20.** ECD spectrum of compound **2**

**
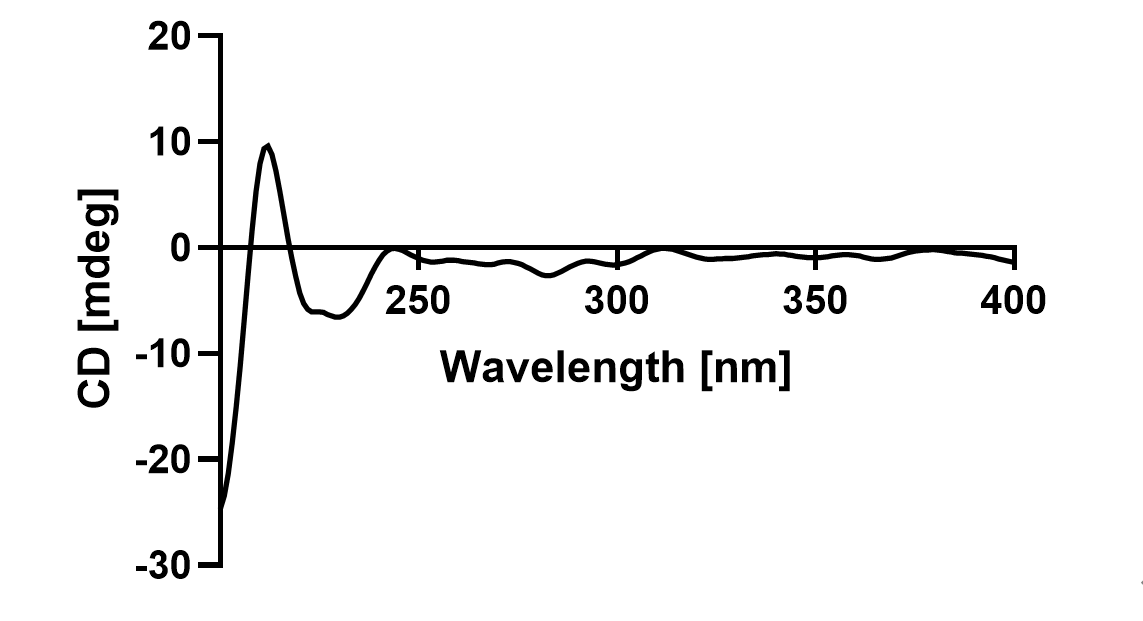
**

**Figure S21.** ^1^H NMR spectrum of compound **3** (Recorded in methanol-*d*_4_)

**Figure S22.** ^13^C NMR and DEPT spectra of compound **3** (Recorded in methanol-*d*_4_)

**Figure S23.** HSQC spectrum of compound **3** (Recorded in methanol-*d*_4_)

**Figure S24.** HMBC spectrum of compound **3** (Recorded in methanol-*d*_4_)

**Figure S25.** ^1^H–^1^H COSY spectrum of compound **3** (Recorded in methanol-*d*_4_)

**Figure S26.** NOESY spectrum of compound **3** (Recorded in methanol-*d*_4_)

**Figure S27.** HRESIMS spectrum of compound **3**

**Figure S28.** UV spectrum of compound **3**


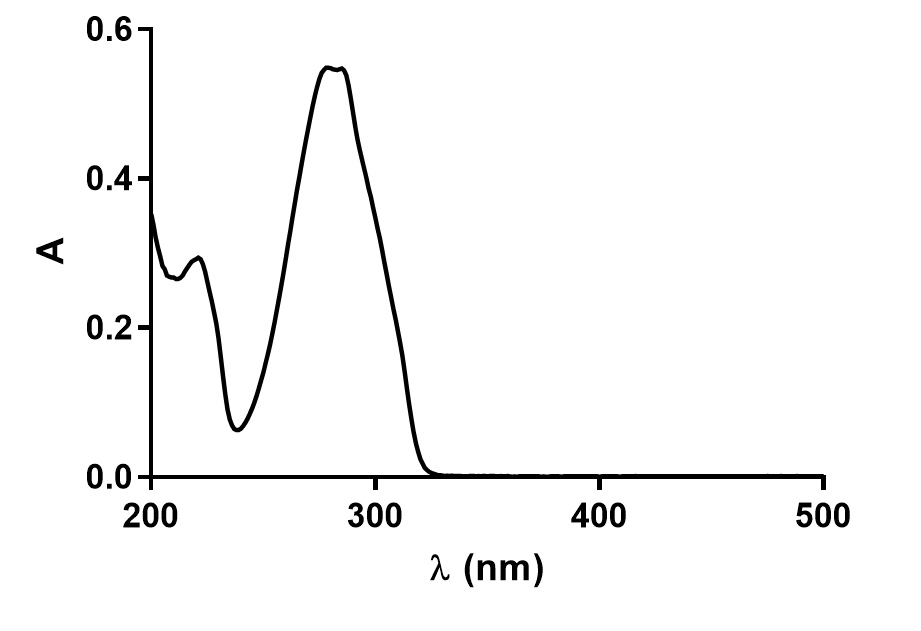


**Figure S29.** IR spectrum of compound **3**

**Figure S30.** ECD spectrum of compound **3**


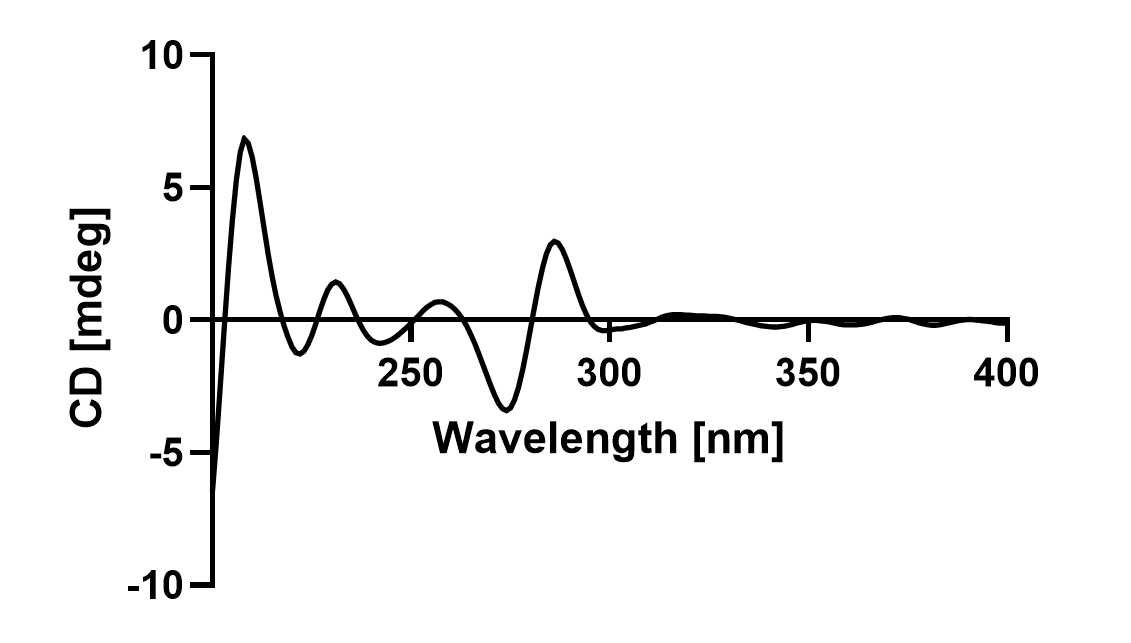


**Experimental**

*1 General*

Optical rotations were measured on a Perkin-Elmer 341 spectropolarimeter in MeOH. UV spectra were collected in MeOH on a Lambda 35 instrument. IR spectra were recorded on a Vertex 70 FT-IR spectrophotometer. ECD data were collected from a JASCO-810 spectrometer. HRESIMS data were obtained from a Bruker MicrOTOF II spectrometer. 1D & 2D NMR spectra were tested on a Bruker AM-400 or AM-600 spectrometer. The chemical shifts (*δ*) were expressed in ppm with reference to the solvent signals for methanol-*d*_4_ (*δ*_H_ 3.31 and *δ*_C_ 49.0). Semipreparative HPLC was performed on an Agilent HPLC system with a reversed-phase (RP) C_18_ column (10 × 250 mm). Column chromatography (CC) was carried out on ODS (50 *μ*m), silica gel (200–300 and 100–200 meshes; Qingdao Marine Chemical Inc., Qingdao, China), and Sephadex LH-20. Fractions were monitored by TLC and spots were visualized by heating silica gel plates sprayed with 10% H_2_SO_4_ in EtOH.

*2*. *Fungal material*

Strain *P. roqueforti* was isolated from the root soil of *Hypericum beanii* N. Robson collected from Shennongjia Forestry District, Hubei Province, China, in August 2018. This strain was authenticated based on the morphology and sequence analysis of the ITS region of rDNA. The sequence data for this strain have been deposited in GenBank with the accession number ON795101. The fungus was preserved in the culture collection of the College of Pharmacy, Tongji Medical College, Huazhong University of Science and Technology.

*3*. *Extraction and isolation*

The fungal strain was cultured on potato dextrose agar (PDA) at 26 °C to prepare the seed cultures. Afterward, the agar plugs were cut into small pieces and inoculated into 240 Erlenmeyer flasks (1 L), each containing 250 g of rice and 180 mL of distilled water, sterilized by autoclave. After incubation at 26 °C for 30 days, the fermented rice substrate was extracted with 95% aqueous ethanol seven times. The organic solvents were evaporated under vacuum to obtain an extract (358 g). The total residue was fractionated by silica gel column chromatography (CC) eluted with petroleum ether (PE)-ethyl acetate (EtOAc) gradient system (30:1→0:1) to obtain six main fractions (A–F) based on TLC analyses. Fraction B (6.8 g) was isolated by RP-C_18_ silica gel CC eluted with MeOH–H_2_O (20%, 40%, 60%, 80%, and 100%) to afford four main fractions (B1–B4). Fraction B2 (1.2 g) was subjected to silica gel CC (PE–EtOAc, 10:1–1:1, v/v) to afford four main subfractions (B2-1–B2-4). Subfraction B2-3 (256 mg) was separated by repeated semipreparative RP-C_18_ HPLC (MeCN–H_2_O, 45:55, v/v; 2.0 mL/min) to afford compounds **1** (8.5 mg, *t*_R_ = 37 min) and **7** (10.0 mg, *t*_R_ = 21 min). Fraction C (35.6 g) was further separated on an ODS column followed by stepwise gradient elution with MeOH–H_2_O (20%–100%) to afford six main subfractions (C1–C6). Subfraction C4 (3.3 g) was applied to silica gel CC using a gradient elution with PE–EtOAc (stepwise 15:1–1:1, v/v), and further purified by semipreparative HPLC (MeOH–H_2_O, 40:60, v/v; 2.0 mL/min) to provide compounds **2** (14.3 mg, *t*_R_ = 35 min) and **4** (21.5 mg, *t*_R_ = 21 min). Subfraction C5 (1.8 g) was repeatedly fractionated via Sephadex LH-20 (CH_2_Cl_2_–MeOH, 1:1, v/v), silica gel CC (PE–EtOAc, 10:1–1:1, v/v), and semipreparative HPLC (MeCN–H_2_O, 35:65, v/v; 2.0 mL/min) to afford compounds **3** (1.6 mg, *t*_R_ = 16 min), **5** (1.0 mg, *t*_R_ = 17 min), and **6** (3.5 mg, *t*_R_ = 25 min).

**Compound 1**: C_29_H_41_NO_7_; colorless block crystals; UV (MeOH) *λ*_max_ (log *ε*): 201 (4.57) and 263 (4.63) nm; IR (KBr) *ν*_max_: 3433, 2929, 2872, 1707, 1660, 1196, 1097, and 1031 cm^–1^; ECD (MeOH) *λ*_max_ (Δ*ε*): 212 (–1.76), 235 (+5.78), and 264 (–2.00) nm; positive HRESIMS *m/z* 538.2791 [M + Na]^+^ (calcd for C_29_H_41_NO_7_Na^+^, 538.2775).

**Compound 2**: C_24_H_25_NO_5_; yellow oil; UV (MeOH) *λ*_max_ (log *ε*): 201 (4.36), 229 (3.96), 278 (3.43), and 349 (3.39) nm; IR (KBr) *ν*_max_: 3442, 2967, 2932, 1698, 1608, 1514, 1437, 1405, 1274, 1176, and 841 cm^–1^; ECD (MeOH) *λ*_max_ (Δ*ε*): 212 (+4.85) and 232 (–3.12) nm; positive HRESIMS *m/z* 430.1631 [M + Na]^+^ (calcd for C_24_H_25_NO_5_Na^+^, 430.1625).

**Compound 3**: C_12_H_14_N_2_O_2_; yellow oil; UV (MeOH) *λ*_max_ (log *ε*): 221 (3.77) and 279 (4.04) nm; IR (KBr) *ν*_max_: 3433, 3310, 2462, 1651, 1636, 1557, 1280, 940, 747, and 695 cm^–1^; ECD (MeOH) *λ*_max_ (Δ*ε*): 209 (+0.88), 223 (–0.15), 231 (+0.19), 242 (–0.12), 257 (+0.09), 274 (–0.45), and 286 (+0.39) nm; positive HRESIMS *m/z* 241.0945 [M + Na]^+^ (calcd for C_12_H_14_N_2_O_2_Na^+^, 241.0947).

*4*. *X-ray crystal structure analysis*

A suitable crystal of **1** was obtained by slow evaporation from MeOH–H_2_O (20:1, v/v) at 4 °C. The intensity data for these compounds were collected with a Bruker APEX DUO diffractometer equipped with an APEX II CCD using graphite-monochromated Cu K*α* radiation. The structures were solved via direct approaches with the SHELXS-97 software package and refined via means of full-matrix least-squares on *F*^2^ [1-2]. Crystallographic data for the reported structures were deposited in the Cambridge Crystallographic Data Center (CCDC) with deposition numbers CCDC 2238093 for **1**. Copies of the data can be obtained free of charge from the CCDC, 12 Union Road, Cambridge CB 1EZ, UK [fax: Int. +44(0) (1223) 336 033); e-mail: [deposit@ccdc.cam.ac.uk](mailto:deposit@ccdc.cam.ac.uk)].

*Crystallographic data for compound* ***1***: C_29_H_41_NO_7_, *M* = 515.63, *a* = 15.7101(9) Å, *b* = 15.7101(9) Å, *c* = 62.953(4) Å, *α* = 90°, *β* = 90°, *γ* = 120°, *V* = 13455.7(18) Å^3^, *T* = 100(2) K, space group *P*65, *Z* = 18, *μ*(Cu K*α*) = 0.660 mm^–1^, 155616 reflections measured, 17064 independent reflections (*R_int_* = 0.0773). The final *R_1_* values were 0.0464 (*I* > 2*σ*(*I*)). The final *wR*(*F*^2^) values were 0.1229 (*I* > 2*σ*(*I*)). The final *R_1_* values were 0.0479 (all data). The final *wR*(*F*^2^) values were 0.1247 (all data). The goodness of fit on *F*^2^ was 1.009. Flack parameter = 0.02(5).

References:

[1] Dolomanov OV, Bourhis LJ, Gildea RJ, Howard JAK, Puschmann H. OLEX2: a complete structure solution, refinement and analysis program. J Appl Crystallogr. 2009;42:339-341.

[2] Sheldrick GM. A short history of SHELX. Acta Crystallogr A. 2008;64:112-122.

**Cytotoxicity and cell apoptosis assay**

*1 Cell viability assay*

Cell viability was measured by the CCK8 kit (Topscience Co., Ltd., China) according to the manufacturer's instructions. Briefly, six human tumor cell lines were placed in 96-well culture plates (3000~6000 per well), processed for 48 h, and then incubated with CCK8 reagent for 4 h at 37 °C. The absorbance at 450 nm was measured using a plate reader (Synergy HT, Biotek). Cell viability was calculated according to the following formula: cell viability (%) = (ODexperiment – ODblank)/(ODcontrol – ODblank) × 100%. All experiments were performed in triplicate. The 50% inhibitory concentration (IC50) was calculated by SPSS software.

*2 Q-PCR analysis*

Total cellular RNA was isolated using TRIzol™ Reagent (Thermo Fisher, USA, Cat: 15596026) and cDNA was synthesized using HiScript® II Q RT SuperMix for qPCR (Vazyme, China, Cat: R223-01). SYBR Green qPCR Mix was purchased from Biosharp (Biosharp, China, Cat: BL698A). β-actin was used for internal reference. The primers used were as follows.

| Gene | Forward primer | Reverse primer |
| --- | --- | --- |
| Gdf15 | CTCAGGACGGTGAATGGCTCTC | CGGAATCTGGAGTCTTCGGAGT |
| SESN2 | ACTCGCTCTCCTCCTTCGTGTT | GTTGTTCAACGGGTCCCTGCTT |
| ATF4 | TTCTCCAGCGACAAGGCTAAGG | CTCCAACATCCAATCTGTCCCG |
| Txnip | CAGCAGTGCAAACAGACTTCGG | CTGAGGAAGCTCAAAGCCGAAC |
| Trib3 | GCTTTGTCTTCGCTGACCGTGA | CTGAGTATCTCAGGTCCCACGT |
| β-actin | AACTACCTTCAACTCCATCA | CTCCTTCTGCATCCTGTC |

*3 Cell cycle analysis*

Cells were treated with vehicle control (DMSO, <0.1%) or compound **1** (10~30 *μ*M) or VP16 (10 *μ*M) for 24 h and then harvested and fixed in 70% ethanol overnight at 4 °C. After washing with cold PBS, the cells were resuspended in 200 *μ*L of PI/Triton X-100 staining solution. Data were acquired using flow cytometry (BD Accuri C6, California, USA). The cell cycle distribution was determined using FlowJo software.

*4 Intracellular ROS determination*

The levels of intracellular ROS after drug treatment were analyzed using the fluorescent probe DCFH-DA kit (Beyotime, China). First, cells were inoculated in 24-well plates at 50000 per well and cells were harvested after incubation with the compound for 24 h. Cells were incubated with 10 *μ*M DCFH-DA for 20 min at 37 °C. The excess probe was washed with PBS and the DCFH fluorescence signal was detected by flow cytometry at an excitation wavelength of 488 nm and an emission wavelength of 585 nm.

*5 Statistical analysis*

Data analysis was performed using GraphPad Prism 8 software. Comparisons between the control and treatment groups were performed. All experiments were repeated at least three times. The data are presented as the mean ± S.D., and *p* values ≤ 0.05 were considered statistically significant. **p* < 0.05, ***p* < 0.01, ****p* < 0.005, *****p* < 0.001.
